# Blood Pressure-Lowering Peptides from Neo-Fermented Buckwheat Sprouts: A New Approach to Estimating ACE-Inhibitory Activity

**DOI:** 10.1371/journal.pone.0105802

**Published:** 2014-09-15

**Authors:** Masahiro Koyama, Seiji Hattori, Yoshihiko Amano, Masanori Watanabe, Kozo Nakamura

**Affiliations:** 1 Department of Bioscience and Biotechnology, Faculty of Agriculture, Shinshu University, Minamiminowa Village, Nagano, Japan; 2 Department of Bioscience and Biotechnology, Graduate School of Agriculture, Shinshu University, Minamiminowa Village, Nagano, Japan; 3 Department of Chemistry and Material Engineering, Faculty of Engineering, Shinshu University, Nagano City, Nagano, Japan; 4 Department of Food, Life and Environmental Science, Faculty of Agriculture, Yamagata University, Tsuruoka City, Yamagata, Japan; 5 Academic Assembly, Institute of Agriculture, Shinshu University, Minamiminowa Village, Nagano, Japan; Universidade Federal do Rio de Janeiro, Brazil

## Abstract

Neo-fermented buckwheat sprouts (neo-FBS) contain angiotensin-converting enzyme (ACE) inhibitors and vasodilators with blood pressure-lowering (BPL) properties in spontaneously hypertensive rats (SHRs). In this study, we investigated antihypertensive mechanisms of six BPL peptides isolated from neo-FBS (FBPs) by a vasorelaxation assay and conventional *in vitro*, *in vivo*, and a new *ex vivo* ACE inhibitory assays. Some FBPs demonstrated moderate endothelium-dependent vasorelaxation in SHR thoracic aorta and all FBPs mildly inhibited ACE *in vitro*. Orally administered FBPs strongly inhibited ACE in SHR tissues. To investigate detailed ACE-inhibitory mechanism of FBPs in living body tissues, we performed the *ex vivo* assay by using endothelium-denuded thoracic aorta rings isolated from SHRs, which demonstrated that FBPs at low concentration effectively inhibited ACE in thoracic aorta tissue and suppressed angiotensin II-mediated vasoconstriction directly associated with BPL. These results indicate that the main BPL mechanism of FBP was ACE inhibition in living body tissues, suggesting that high FBP's bioavailability including absorption, tissue affinity, and tissue accumulation was responsible for the superior ACE inhibition *in vivo*. We propose that our *ex vivo* assay is an efficient and reliable method for evaluating ACE-inhibitory mechanism responsible for BPL activity *in vivo*.

## Introduction

The main target of the pharmaceutical industry for antihypertensive drugs remains angiotensin-converting enzyme (ACE). This transmembrane metalloprotease catalyzes the conversion of angiotensin (Ang) I to a potent vasoconstrictor Ang II [1]. This signaling molecule binds AT1 receptors on endothelial cells and vascular smooth muscle cells (SMCs) to promote a variety of functions, including vasoconstriction, tissue remodeling, and inflammation [2]. ACE expression in blood vessels is normally limited to endothelial cells [3]; however, hypertension and atherosclerosis are associated with ACE expression on human endothelial cells, subendothelial neointimal cells, and SMCs [4]. Likewise, spontaneously hypertensive rats (SHRs), an animal model of human essential hypertension, functionally express ACE on endothelial cells and SMCs [5]. ACE expression on SMCs is largely responsible for hypertension via vasoconstriction mediated by excessive production of Ang II [4]. Accordingly, ACE inhibitors are routinely prescribed to patients with hypertension [6]. They reduce blood pressure and partially correct vascular remodeling by suppressing Ang II production by all ACE-expressing cell types, including SMCs.

Many peptidic ACE inhibitors with blood pressure-lowering (BPL) effects have been identified in antihypertensive foods [7], [8]. To induce *in vivo* BPL action via ACE inhibition, the inhibitory peptides must reach and remain in the target tissue in their active form. It has been reported that short-chain peptides are absorbed in the blood in relatively intact form [9]. However, many proteases and peptidases present in living body tissues may degrade the absorbed peptides converting them to inactive forms [10]. Therefore, bioavailability including absorption, tissue affinity, and tissue accumulation as well as binding affinity of ACE inhibitory peptides to ACE are important factors for ACE inhibition and subsequent induction of BPL activity *in vivo*.

In the previous study, we developed a new antihypertensive food called neo-fermented buckwheat sprouts (neo-FBS) by lactic bacteria fermentation of buckwheat sprouts [11]. This extract had potent lowering effects on systolic blood pressure (SBP) and diastolic blood pressure (DBP) in SHRs after a single oral dose of 0.01 mg/kg body weight (BW). The extract inhibited tissue ACE activity and evoked vasorelaxation of phenylephrine pre-constricted aortic rings in SHRs. We reported that both activities were responsible for BPL action [11]. Neo-FBS contained six novel BPL peptides: DVWY, FDART, FQ, VAE, VVG, and WTFR [12]. These peptides significantly reduced SBP and DBP in SHRs after single oral administration of 0.1 mg/kg. Although it was thought that vasorelaxation and/or ACE inhibition *in vivo* were responsible for BPL effects of these peptides, which were similar to the effects of neo-FBS; their specific BPL mechanisms remained unclear.

In this study, we investigated the BPL mechanisms of these six BPL peptides isolated from neo-FBS (FBPs) by measuring vasorelaxation and ACE-inhibitory activity. Vasorelaxation was measured using the SHR thoracic aorta ring assay. The ACE-inhibitory activity was measured by the *in vitro* and *in vivo* assays generally used to evaluate food-derived peptidic ACE inhibitors. In addition, we performed a new *ex vivo* ACE-inhibitory assay to investigate detailed BPL mechanism based on ACE inhibition by FBPs in living body tissue. The *ex vivo* assay measured FBP effects on ACE expressed on SMCs by using endothelium-denuded thoracic aorta ring of SHRs. Finally, we discussed the ACE-inhibitory properties of FBPs based on the *in vitro*, *ex vivo*, and *in vivo* results.

## Materials and Methods

### Animals and ethics statement

All animal experiments were performed with 10–13 week-old male SHRs (SHR/NCrlcrlj). Housing and feeding was conducted as previously described [11]. All animal experiments were carried out in strict accordance with the recommendations of the Standards Relating to the Care and Management of Laboratory Animals and Relief of Pain (2006) published by the Japanese Ministry of the Environment. The protocol was approved by the Animal Care Committee of the Faculty of Agriculture of Shinshu University (permit number: 230066). Surgery was performed under diethyl ether anesthesia, and all efforts were made to minimize suffering.

### Chemicals

Acetylcholine chloride (ACh), Ang I (>97%), Ang II (>97%), captopril, and phenylephrine hydrochloride (PE) were purchased from Wako Pure Chemical Industries (Osaka, Japan). ACE (EC 3.4.15.1) purified from the rabbit lungs was obtained from Sigma-Aldrich Japan K.K. (Tokyo, Japan). Hippuryl-l-histidyl-l-leucine (Hip-His-Leu) was obtained from the Peptide Institute (Osaka, Japan). Ammonium formate, borate, diethyl ether, ethyl acetate, formate, hydrochloric acid (HCl), HPLC-grade acetonitrile, sodium chloride (NaCl), potassium chloride (KCl), potassium dihydrogen phosphate (KH_2_PO_4_), magnesium acetate (Mg(CH_3_COO)_2_), magnesium sulfate (MgSO_4_), Nonidet P-40, sodium hydrogen carbonate (NaHCO_3_), sucrose, and tris(hydroxymethyl)aminomethane were purchased from Kanto Chemical Co., Inc. (Tokyo, Japan). Trifluoroacetic acid (TFA) was purchased from Watanabe Chemical Industries, Ltd. (Hiroshima, Japan).

### Preparation of FBPs

Six FBPs (DVWY, FDART, FQ, VAE, VVG, and WTFR) were recently identified in neo-FBS by HPLC analysis, LC-MS/MS, and Edman degradation [12]. These FBPs were synthesized and purified to high degree (>95%) in our laboratory as described [12].

### Measurement of vasorelaxant effect in SHR thoracic aorta rings

The effects of FBPs were measured using thoracic aorta isolated from SHRs (n = 6). Isolation of the thoracic aorta and preparation of the aortic rings (2–3 mm long) were performed as described previously [11]. In some rings, the endothelium was removed by gentle rubbing of the intimal surface with small forceps. The rings were mounted in an organ bath at 37°C in Krebs solution (119 mM NaCl, 4.7 mM KCI, 1.1 mM KH_2_PO_4_, 1.2 mM MgSO_4_, 25 mM NaHCO_3_, pH 7.4) bubbled with 95% O_2_/5% CO_2_ and stretched to a resting tension of 1.5 g. Equilibration of the tension, confirmation of the endothelium function by Ach, and stabilization of the vasoconstrictive force by PE were performed as previously described [11]. After this pretreatment, the tissues were constricted again with PE. When stable constriction was obtained, each FBP was dissolved in Krebs solution and were cumulatively added to the bath at final concentrations of 0.50, 1.0, 5.0, 10, 25, 50, 75, and 100 µg/mL. This concentration range is based on the results of our previous study where neo-FBS induced significant vasorelaxation in the aorta assay [11]. Vasorelaxant effect was expressed as the percentage of reduction of PE-induced constriction. The data are presented as means ± S.E. (n = 3).

### Measurement of *in vitro* ACE-inhibitory activity

The *in vitro* ACE inhibition assay was performed using the method described previously [11]. Briefly, each FBP was prepared at three final concentrations determined in preliminary experiments: 0.17, 0.86, and 4.3 mM for DVWY; 0.66, 3.3, and 16 mM for FDART; 6.8, 14, and 27 mM for FQ; 63, 126, and 252 mM for VAE; 3.7, 7.3, and 15 mM for VVG; and 1.6, 8.2, and 41 mM for WTFR. Each solution (30 µL) was mixed with the ACE substrate (7.6 mM Hip-His-Leu) in borate buffer (0.10 M borate and 0.60 M NaCl, pH 8.3) and incubated at 37°C for 7 min. Then, 100 µL of commercial ACE (6.0 mU) was added, and the mixture was incubated at 37°C for 30 min. The reaction was stopped by 250 µL of 1.0 N HCl. Released hippuric acid (Hip) was extracted with 1.0 mL ethyl acetate and quantified by HPLC according to our previous report [11]. A linear Hip calibration curve was obtained in the concentration range of 5.0–50 µg/mL with a correlation coefficient of 0.9995; linearity is described by the equation y = 27319x+200598. *In vitro* ACE-inhibitory activity (%) was calculated using the equation described previously [11] and expressed as the 50% inhibitory concentration (IC_50_). All measurements were performed in triplicate for each sample concentration, and the results were expressed as the mean ± S.E.

### Measurement of *in vivo* ACE-inhibitory activity

Animals were assigned to six FBP-treated groups and six control group; the average blood pressure was the same in each group. After being deprived of food for 12 h, rats were given an oral dose of 10 mg/kg BW FBP in purified water (n = 6) by gastric intubation. The dose, which was higher than the minimum effective BPL dose of FBP (0.1 mg/kg BW), had been estimated based on ACE-inhibitory activity *in vitro* and BPL activity *in vivo*, of a food derived di-peptide Val-Tyr (VY) that affects renin-angiotensin system. This system plays an important role in the regulation of blood pressure and body fluid homeostasis, and VY is known as an ACE inhibitor with BPL activity [8] and a body fluid regulator via reactivation of Ca^2+^-ATPase inhibited by Ang II [13]. The *in vitro* ACE-inhibitory activity (IC_50_) and minimum effective BPL dose of VY were 26.0 µM and 1.0 mg/kg, respectively [8]. Among FBPs, the most potent *in vitro* ACE inhibitor was DVWY (690 µM); based on this concentration, the minimal *in vivo* dosage of FBPs for effective BPL via ACE inhibition was estimated as 10 mg/kg, which is consistent with the concentration of neo-FBS that induced a significant *in vivo* ACE inhibition in our previous study [11].

Prior to and 6 h after the administration, SBP and DBP were measured in all SHRs by the tail-cuff method [11]. Isolation and preparation of the tissue and plasma samples were performed as previously reported [11]. Briefly, the thoracic aortas, hearts, livers, kidneys, and lungs isolated from the test animals were homogenized at 0°C in 50 mM Tris-HCl buffer (3.0 mM KCl, 0.50 mM Mg(CH_3_COO)_2_, 25 mM sucrose, 50 mM tris(hydroxymethyl)aminomethane, 0.50% Nonidet P-40, pH 7.8) for 5 min (heart, liver, kidney, and lungs) or 10 min (thoracic aorta). The homogenates were centrifuged at 12,000×*g* for 20 min at 4°C, and the supernatant was immediately used to measure tissue ACE activity. Plasma samples were prepared by centrifugation (3,200×*g*, 20 min, 4°C) of the blood collected in lithium heparin-treated tubes. Tissue and plasma ACE activity was measured as described for the *in vitro* assay and expressed as units/mg-protein or units/mL, respectively. Tissue protein content was measured by the BCA Protein Assay Reagent Kit (Thermo Fisher Scientific K.K., Kanagawa, Japan). The results were expressed as the mean ± S.E.

### Measurement of Ang II production and Ang II-mediated vasoconstriction in SHR thoracic aorta rings by *ex vivo* ACE inhibition assay

Our *ex vivo* assay measured both Ang II production and Ang II-mediated vasoconstriction in endothelium-denuded thoracic aorta rings isolated from SHRs; the results were used to compare the effects of different FBPs and captopril on tissue ACE activity. Isolation of the thoracic aorta from SHRs (n = 12) and preparation of endothelium-denuded aortic rings was performed as described above. Endothelium was removed to eliminate the influence of endothelium-derived vasodilators. Prostanoids and nitric oxide, which are major endothelium-derived vasodilators, were shown to strongly suppress vasoconstriction [14]. Therefore, it is difficult to directly measure the ACE inhibition-mediated suppression of vasoconstriction using ACE inhibitors affecting the endothelium such as FBPs.

Before the experiment, the influence of endothelial removal on Ang II production was evaluated using endothelium-intact and endothelium-denuded aortic rings by a procedure similar to that for the negative control described below.

Four FBPs (DVWY, FDART, FQ, and WTFR) with significant thoracic aorta ACE-inhibitory activity in the *in vivo* assay were tested because this assay evaluates tissue ACE-inhibitory activity in thoracic aorta rings. Captopril and Krebs solution were used as the positive and negative controls, respectively. After equilibrating and stabilization of vasoconstriction in aorta rings by PE, 2.0 mL of Krebs solution was removed from the organ bath to add test sample and Ang I. Then, 1.0 mL of DVWY, FDART, FQ, WTFR, captopril, or Krebs solution was added to endothelium-denuded ring tissues to reach final concentrations of 0.1, 1.0, or 10.0 µM for FBPs and 0.1, 0.5, or 1.0 µM for captopril; 1.0 mL of Krebs solution was added to endothelium-intact ring tissues; all the concentrations were determined in preliminary experiments. No significant vasoconstriction or vasodilation of the aorta rings was caused by FBPs. After 30 min incubation, 1.0 mL of 0.20 µM Ang I was added and the aortic tension was recorded. When vasoconstriction plateaued, Krebs solution in the organ bath was transferred to a test tube and heated at 90°C for 5 min to stop the enzyme reaction; the heat-treated samples were dried under vacuum and frozen at −80°C until UPLC-MS analysis. The aorta rings were used in the BCA assay to express Ang II production as pmol/mg-protein. To evaluate FBP suppressive effect on Ang II-mediated vasoconstriction, the tension was recorded and expressed as the percentage of maximum constriction of the negative control; the 50% effective concentration (EC_50_) was determined by plotting FBP and captopril concentrations against the percent of suppression.

### Measurement of *ex vivo* ACE-inhibitory activity by quantification of Ang II by UPLC-MS

UPLC-MS analysis was performed to quantify Ang II produced from Ang I in thoracic aorta treated with FBPs or captopril. Vacuum-dried samples were dissolved in 500 µL acetonitrile/4.0 mM ammonium formate (5∶95 v/v) with 4.0 mM formic acid prior to UPLC-MS analysis, performed at the CREFAS (Collaborated Research Center for Food Functions, Faculty of Agriculture, Shinshu University); Quattro micro API (MS) with an ACQUITY UPLC (Waters Co., USA) was used. Separation was conducted at 35°C using a CHEMCOBOND 5-ODS-W reversed-phase column (4.6×150 mm; ChemcoPlus Scientific Co., Ltd. Kyoto, Japan). Elution was performed at 0.80 mL/min using acetonitrile/4.0 mM ammonium formate (5∶95 v/v) with 4.0 mM formic acid (solvent A) and acetonitrile/4.0 mM ammonium formate (90∶10 v/v) with 4.0 mM formic acid (solvent B): 0–2 min, isocratic 0% solvent B; 2–10 min, 0–40% solvent B; 10–15 min, isocratic 40% solvent B; 15–20 min, 40–90% solvent B. UV detection was performed at 215 nm with an injection volume of 20 µL. Mass spectra were acquired in electrospray ionization (ESI) mode using 3500 V capillary voltage, 30 V cone voltage, 350 L/h N_2_ gas flow (desolvation), 50 L/h N_2_ gas flow (cone), 100°C source temperature, and 350°C desolvation temperature. The mass spectrometer was operated in positive mode and selected ion monitoring (SIM) mode at *m/z* 523.8 [M+2H]^2+^ for Ang II. Ang II concentration was calculated using standard calibration curves in the linear range of 0.0010–1.0 µM with a correlation coefficient of 0.9993; linearity was described by the equation y = 69455x+423.12. The IC_50_ of Ang II production was determined by plotting FBP and captopril concentration versus inhibition of Ang II production calculated as the percentage of negative control group. For each sample, measurements were performed in triplicate; the results were expressed as the mean ± S.E.

### Statistical analysis

All results are expressed as mean ± S.E. The *in vitro* ACE-inhibitory activities of FBPs were analyzed by one-way analysis of variance (ANOVA) followed by Tukey's test. We assessed correlation between the suppression of Ang II production and Ang II-mediated vasoconstriction to validate the *ex vivo* ACE inhibition assay. For this, Pearson's correlation analysis widely applied in studies of enzyme inhibitory activity, was used. A coefficient of correlation (*r*) value >0.70 and *P* value (two-sided test) <0.05 were considered statistically significant. In all animal tests, Student's *t*-test was used to identify significant differences between each FBP group and control group (*p<0.05, **p<0.01).

## Results

In our previous study, we found that the BPL effect of neo-FBS *in vivo* is underlined by vasorelaxation and ACE inhibition [11]. Here, we examined whether these effects are responsible for BPL activity of FBPs: DVWY, FDART, FQ, VAE, VVG, and WTFR.

### Vasorelaxant effects of FBPs

**Figure 1:**
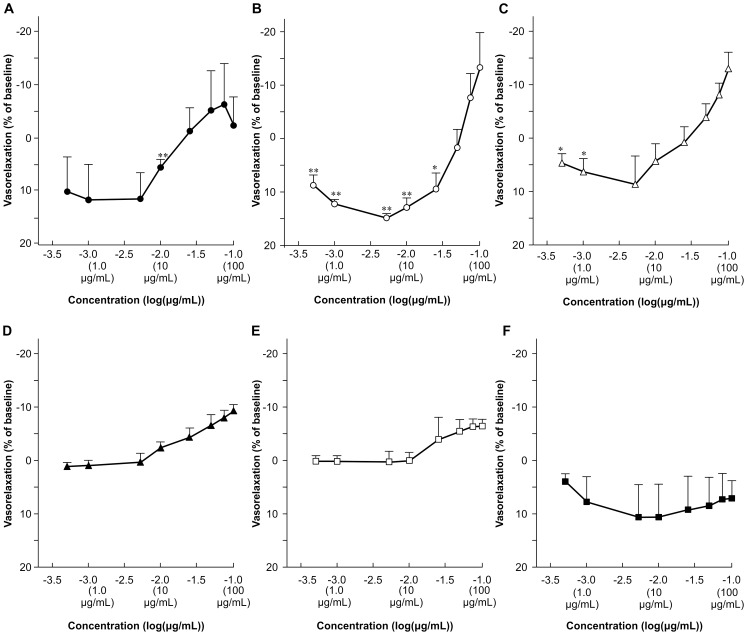
FBP concentration-vasorelaxation curves of phenylephrine-preconstricted thoracic aorta rings from SHRs. Dose dependent response to cumulatively increasing concentration of DVWY (A), FDART (B), VVG (C), FQ (D), VAE (E), and WTFR (F). Vasorelaxant rate is expressed as the percentage reduction of evoked contraction. Each data point and bar represent the mean ± S.E. (n = 3). *p<0.05, **p<0.01, versus pre-treatment tension, Student's *t*-test.

Vasorelaxant effects of FBPs were measured using PE-preconstricted endothelium-intact or endothelium-denuded thoracic aorta rings of SHRs (n = 3). DVWY, FDART, and VVG significantly dilated the endothelium-intact aorta rings (Fig. 1A–C), while FQ, VAE, and WTFR at 0.50 to 100 µg/mL had no significant effects (Fig. 1D–F). FDART and VVG exerted significant vasorelaxation starting at 0.50 µg/mL, and DVWY exerted the effect at 10 µg/mL. FDART, DVWY, and VVG caused maximum vasorelaxation of 14.9±0.4% at 5.0 µg/mL, 11.7±6.5% at 1.0 µg/mL, and 8.2±4.8% at 5.0 µg/mL, respectively; however, vasorelaxation was not observed in endothelium-denuded thoracic aorta rings. These data indicate that some FBPs have the capacity to dilate PE-preconstricted thoracic aorta rings of SHRs and that the vasorelaxation depended on the endothelium.

### *In vitro* ACE-inhibitory activities of FBPs

FBPs were tested in a standard *in vitro* assay with Hip-His-Leu substrate. Table 1 shows that all tested peptides inhibited ACE activity *in vitro* (n = 3). Based on the IC_50_ values, the most potent FBP was DVWY (IC_50_, 0.69±0.04 mM), followed by FDART (1.9±0.1 mM), WTFR (6.7±0.5 mM), FQ (7.4±0.6 mM), VVG (39.6±5.7 mM), and VAE (55.9±1.9 mM). These experiments revealed significant variability among FBPs (ANOVA: F = 62.6, *p* = 1.24×10^−8^).

**Table 1:** *In vitro* ACE-inhibitory activities (IC_50_) of FBPs.

	DVWY	FDART	FQ	VAE	VVG	WTFR
IC_50_(mM)	0.69±0.04^a^	1.9±0.1^a^	7.4±0.6^a^	55.9±1.9^b^	39.6±5.7^b^	6.7±0.5^a^

Data represent the mean ± S.E. (n = 3). Different letters indicate significant differences between each FBP (*p*<0.05, one-way ANOVA followed by Tukey's test).

### *In vivo* ACE-inhibitory activities of FBPs

**Figure 2:**
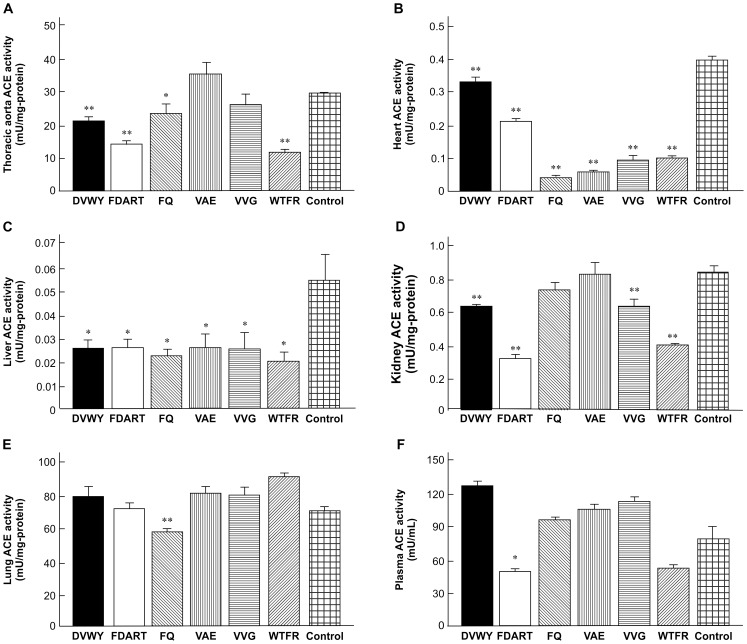
Tissue and plasma ACE activities in SHRs after treatment with FBPs. Animals were treated with FBPs at a dose of 10 mg/kg BW. The levels of ACE activity in thoracic aorta (A), heart (B), liver (C), kidney (D), lung (E), and plasma (F) were determined 6 hours after administration. Data represent the mean ± S.E. (n = 6). *p<0.05, **p<0.01 versus the control group (purified water administration), Student's *t*-test.

We examined the *in vivo* effects of FBPs on ACE inhibition at a dosage based on the *in vitro* activity (10 mg/kg BW). Although the dose was higher than the effective BPL dose of FBPs (0.1 mg/kg BW), it was lower than that estimated based on FBP weak *in vitro* ACE-inhibitory activity. SHRs were treated with each FBP and ACE activity was measured in the thoracic aorta, heart, liver, kidney, lung, and plasma 6 h after FBP administration (n = 6, Fig. 2). FBPs strongly inhibited tissue ACE in the circulatory system organs, including thoracic aorta and heart. In the aorta, all FBPs except for VAE and VVG, significantly decreased ACE activity by 21–61% compared with the control group; WTFR was the most efficient among the FBPs. In the heart, all FBPs caused significant inhibitory activity of 17–91%; FQ was the most efficient ACE inhibitor. ACE activity in the liver and kidney were also significantly inhibited by most FBPs and decreased by 53–64% and 25–63%, respectively. In addition, moderate ACE inhibitory activity was exhibited by FQ and FDART in the lungs and plasma.

All *in vivo* ACE-inhibiting FBPs significantly reduced blood pressure in SHRs at 6 h post-administration (Table 2). FBP caused stronger reduction of SBP than of DBP. Overall, these data show that all FBPs at 10 mg/kg effectively inhibited tissue and plasma ACE and exerted BPL activity.

**Table 2:** Maximum systolic and diastolic blood pressure pressure-lowering values at 6 h after administration of FBPs to SHRs (10 mg/kg BW, n = 6).

	DVWY	FDART	FQ	VAE	VVG	WTFR
SBP (mmHg)	−28.3±5.0**	−24.7±3.4**	−27.3±8.3*	−15.8±4.7*	−20.1±5.5**	−21.1±7.1*
DBP (mmHg)	−19.2±5.2*	−16.9±7.6	−18.6±8.5*	−15.8±3.8*	−17.0±5.8*	−16.6±7.5

* *p*<0.05,

** *p*<0.01, versus the negative control group as evaluated by Student's *t*-test.

### *Ex vivo* ACE-inhibitory activities of FBPs

**Figure 3:**
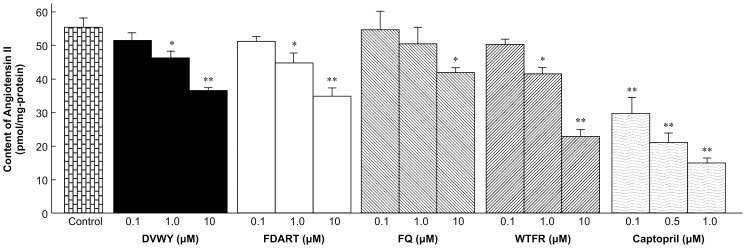
Angiotensin II production after incubation with angiotensin I and DVWY, FDART, FQ, WTFR, and captopril. Angiotensin II production was measured in SHR aorta rings incubated in 0.2 µM angiotensin I with DVWY, FDART, FQ, WTFR, and captopril (positive control). Data represent the mean ± S.E. (n = 3). *p<0.05, **p<0.01 versus the negative control group (Krebs solution treatment), Student's *t*-test.

To examine specific ACE-inhibitory mechanisms of FBPs in living body tissues and their contribution to BPL activity, we designed a new *ex vivo* assay based on endothelium-denuded thoracic aorta rings of SHRs. The four most potent peptides identified in the *in vitro* and *in vivo* assays, DVWY, FDART, FQ, and WTFR, and captopril as positive control were examined. First, we tested the effect of endothelium removal on Ang II production by incubating endothelium-denuded aorta rings with Ang I (n = 3). There was no significant difference in Ang II production between endothelium-denuded (55.5±3.5 pmol/mg-protein) and endothelium-intact samples (67.6±6.4 pmol/mg-protein), indicating that in thoracic aorta of SHRs, ACE is mainly expressed and acted on mesothelium. Thus, the endothelium-denuded aorta rings could be used to measure ACE-inhibitory activity of FBPs in thoracic aorta. Figure 3 shows that all four FBPs significantly reduced Ang II production in a dose-dependent manner, starting at 1.0 µM (DVWY, FDART, and WTFR) or 10 µM (FQ) (n = 6). The most potent peptide was WTFR, which reduced Ang II production by 59% at 10 µM. Captopril also efficiently reduced Ang II production at 0.10 µM. The IC_50_ values were 0.15 µM (captopril), 5.64 µM (WTFR), 41.7 µM (FDART), 70.5 µM (DVWY), and 90.9 µM (FQ). These data suggest that FBPs effectively inhibited ACE in thoracic aorta at much lower concentrations than indicated by their *in vitro* activity (IC_50_).

**Figure 4:**
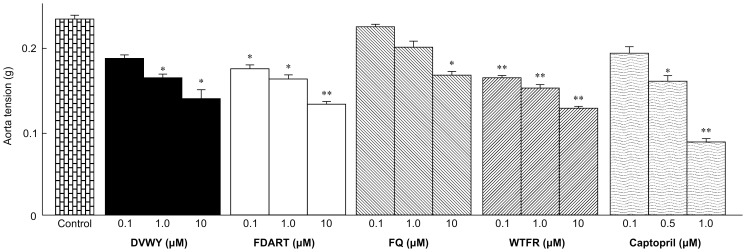
Vascular tension changes after incubation with angiotensin I and DVWY, FDART, FQ, WTFR, and captopril. Tension changes were measured using SHR thoracic aorta rings incubated in 0.2 µM angiotensin I with DVWY, FDART, FQ, WTFR, and captopril (positive control). Data represent the mean ± S.E. (n = 3). *p<0.05, **p<0.01 versus the negative control group (Krebs solution treatment) by Student's *t*-test.

In our new quantitative *ex vivo* ACE inhibition assay, changes in vascular tone were also measured. Figure 4 shows maximum vasoconstriction of SHR aorta rings incubated with Ang I in the presence of DVWY, FDART, FQ, WTFR, and captopril (n = 6). FBPs significantly suppressed vasoconstriction in a dose-dependent manner, starting at 0.10 µM (FDART and WTFR), 1.0 µM (DVWY), or 10 µM (FQ). Captopril starting from 0.50 µM also suppressed vasoconstriction. The EC_50_ values were 0.75 µM (captopril), 36.8 µM (WTFR), 53.6 µM (FDART), 68.2 µM (DVWY), and 85.1 µM (FQ). These results confirmed that the decrease in Ang II production caused by FBPs suppressed the contractile response of SHR rings. Thus, we confirmed by the new *ex vivo* assay that in the thoracic aorta, FBPs inhibited ACE and suppressed vasoconstriction at low concentrations.

**Figure 5:**
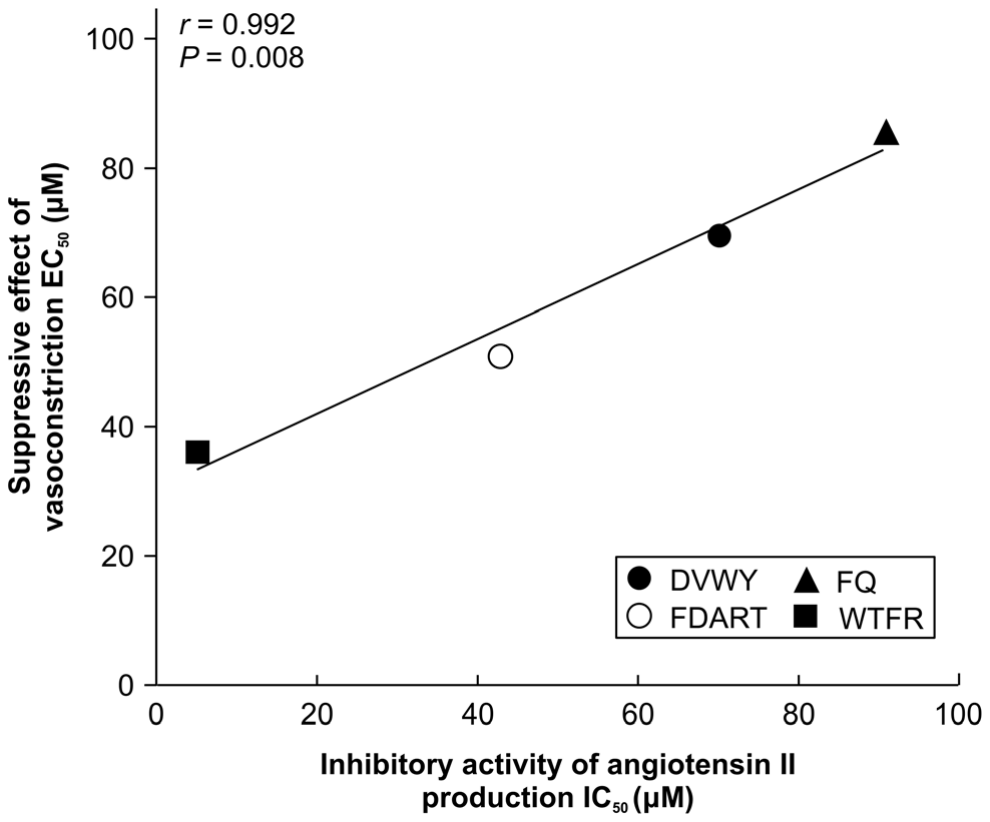
Correlation between suppression of Ang II production and vasoconstriction by DVWY, FDART, FQ, and WTFR. Correlation analysis of suppressive effects of Ang II production (IC_50_) and vasoconstriction (EC_50_). Correlation coefficients (*r*) and two-tailed *P* values are given in the inserts.

The four FBPs reduced Ang II production by inhibiting ACE and suppressed Ang II-mediated vasoconstriction, thus triggering reduction in blood pressure. However, we could not conclude that the suppression of vasoconstriction was caused only by Ang II decrease. Although FBPs did not affect the endothelium-denuded PE-constricted thoracic aorta, they might demonstrate other vasoactive effects such as inhibition of Ang II binding to the AT1 receptor. To confirm that ACE inhibition was the major factor causing the suppression of vasoconstriction by FBPs, Pearson's correlation analysis was performed for ACE-inhibitory activity (IC_50_) as variable ‘x’ and suppression of vasoconstriction (EC_50_) as variable ‘y’ (Fig. 5). The IC_50_ values showed significant positive correlation with EC_50_ (*r* = 0.992; *P* = 0.008), indicating that the decrease in Ang II was directly related to the suppression of vasoconstriction without any interfering factors. Given that suppression of vasoconstriction results in BPL [15], the correlation between suppression of Ang II production and vasoconstriction demonstrated that Ang II inhibition was responsible for BPL. Thus, the *ex vivo* results suggest that ACE inhibition by FBPs in the thoracic aorta effectively reduced blood pressure by suppressing Ang II-mediated vasoconstriction.

In conclusion, our results demonstrate that the main mechanism of FBP-related BPL activity is the inhibition of ACE production in living body tissues.

## Discussion

To characterize BPL effects of six peptides identified in neo-FBS, we employed a vasorelaxation assay, traditional *in vitro* and *in vivo* ACE inhibition assays, and a new *ex vivo* ACE inhibition assay.

FDART and VVG induced significant endothelium-dependent vasorelaxation in doses starting from 0.50 µg/mL and DVWY did that at 10 µg/mL. However, the effects were considerably weaker than those of neo-FBS. Neo-FBS at 50 µg/mL increased vasodilation of PE-preconstricted SHR thoracic aorta rings by 82% under the same experimental conditions [11], suggesting that the significant BPL effects of orally administered FBPs are not primarily mediated by endothelium-dependent vasorelaxation. Therefore, we measured ACE inhibition which is another BPL mechanism activated by neo-FBS.

All FBPs showed ACE-inhibitory activity *in vitro*; the most potent peptide was DVWY (IC_50_ of 690 µM). Other BPL peptides IPP, VPP, and VY also inhibited ACE (IC_50_ of 5.17 µM, 8.89 µM, and 26.0 µM, respectively) [7], [8]; these are active compounds of Food for Specified Health Uses (FOSHU), which are the products with high blood pressure-modulatory effects (https://hfnet.nih.go.jp/). Our results revealed that the *in vitro* activity of FBPs was much weaker than that of reported peptidic ACE inhibitors. Therefore, we measured the *in vivo* ACE-inhibitory activity of FBPs in SHRs treated with a dose of 10 mg/kg based on the concentration active *in vitro*.

FBPs strongly inhibited ACE in the thoracic aorta, heart, and liver. It has been shown that reduction of ACE activity in the major vessels of the circulatory system, including the thoracic aorta and heart, causes significant BPL [15], [16], whereas in the liver, it prevents fibrosis [17]. We have reported that in SHRs, neo-FBS reduced ACE activity by 36.4% in the thoracic aorta, 61.6% in the heart, 60.6% in the liver, 16.1% in the kidney, and 21.9% in the lungs after a single oral dose of 10 mg/kg [11]. The *in vivo* ACE-inhibitory activity of FBPs was the same level or greater than that of neo-FBS, indicating that FBP inhibitory activity was sufficient to cause BPL in SHRs. Indeed FBPs significantly reduced blood pressure in SHRs, especially SBP (Tab. 2). Previous studies have reported that ACE inhibition in the circulatory system mainly decreased SBP because of Ang II-induced suppression of vasoconstriction [15]. SHRs are widely employed as a model of human essential hypertension, and the effects confirmed in the rats can be extrapolated to humans [18]. Therefore, it was expected that FBPs could effectively reduce blood pressure in hypertensive states via inhibition of ACE activity in living body tissues.

We hypothesized that FBPs could effectively inhibit ACE *in vivo* despite their weak *in vitro* activity. To clarify the ACE-inhibitory mechanism *in vivo*, we performed the *ex vivo* assay. The results demonstrated that FBPs effectively and directly suppressed Ang II-mediated vasoconstriction by inhibiting ACE in the thoracic aorta. Based on the *ex vivo* results, we conclude that FBP effectively reduced blood pressure by inhibiting living body tissue ACE. The endothelium-denuded thoracic aorta rings of SHRs was employed in the *ex vivo* assay, because ACE expressed on SMCs have a major role in thoracic aorta of SHR [19]. And the vasoconstriction suppression by FBPs in the assay was clearly observed without interference of endothelium and this result supports this conclusion. Several *ex vivo* assays of non-peptidic ACE inhibitors in the thoracic aorta have been previously described [20]–[23]. Olszanecki et al. evaluated tissue ACE inhibition based on the reduction of Ang II production using endothelium-intact aortas from Wister-Kyoto (WKY) rats [23]. Other studies evaluated tissue ACE inhibition based on the reduction of vasoconstriction using endothelium-denuded aortas of WKY rats and endothelium-intact aortas of SHRs [20]–[22].

However, we could not have estimated the main BPL mechanism of FBPs by these assays. Response of the endothelium-intact aortas can be caused by the other factors of ACE and the removal of endothelium may have an effect on ACE activity in thoracic aorta of normotensive WKY rat. The use of endothelium-denuded thoracic aorta rings of SHRs could evaluate proper tissue ACE-inhibitory activity of FBPs. Thus, the *ex vivo* assay which has not yet been reported may be useful to examine the activity of peptidic ACE inhibitor in hypertensive state under the similar conditions *in vivo*.

The *in vitro*, *in vivo*, and *ex vivo* results demonstrated that ACE-inhibitory activity of FBPs was very high in living body tissues despite weak *in vitro* responses. To investigate this discrepancy, we compared the inhibitory activity of WTFR, which demonstrated the strongest tissue ACE inhibition among FBPs, with that of captopril. Captopril is an effective orally administered ACE inhibitory drug widely used in treating hypertension. The *in vitro* ACE-inhibitory activity of WTFR (IC_50_ 6.7 mM) was approximately 73,000 times weaker than that of captopril (IC_50_ 0.092 µM) [11], whereas the *ex vivo* WTFR activity (IC_50_ 5.64 µM) was only 37.6 times weaker compared to captopril (IC_50_ 0.15 µM) and the *in vivo* activity of WTFR was similar to that of captopril [11]. A considerable difference between the activity of captopril and FBPs *in vitro* dwindled in the *ex vivo* assays and disappeared *in vivo*. The *in vitro* assay indicated that captopril bound ACE at a much lower concentration than FBPs. Although captopril IC_50_ values *in vitro* and *ex vivo* were comparable, FBP IC_50_ values *ex vivo* were much lower than *in vitro*. Tissue ACE is mainly expressed on the plasma membranes of SMCs in hypertensive state [24]. The inhibitors function after reaching an effective concentration in living body tissues, and it has been reported that peptidic ACE inhibitors showed strong tissue ACE inhibitory activity by accumulating in the target tissues [25,26]. In our *ex vivo* assay, the incubation time of 10 min, which is one-third of proper incubation time (30 min), has waned the ACE-inhibitory activity of FBPs. The presence of FBPs, including WTFR, on thoracic aorta was qualitatively confirmed by UPLC-MS analysis using tissue homogenate prepared from the aorta rings after the end of the *ex vivo* assay (data not shown). By the *ex vivo* assay, we show that FBPs with higher tissue affinity might accumulate in the target organs until they can reach the threshold necessary for ACE inhibition at the level of captopril. In addition to tissue affinity, the *in vivo* ACE-inhibitory activity of FBPs is driven by high absorbability suggesting more efficient accumulation in target tissues compared to captopril. Considering all these factors, we hypothesize that high FBP bioavailability underlines their significant inhibitory effects on tissue ACE comparable to those of captopril.

Captopril is an effective medicine for cardiovascular diseases such as hypertension and heart failure. However, it strongly inhibits ACE in the lungs causing dry cough as a side effect. In addition, it is difficult to use captopril as a hypertension-preventive agent because its application is generally limited to the diseased patients. FBPs are constituents of neo-FBS which is lactic bacteria-fermented food prepared from buckwheat sprouts; the majority of tested FBPs (except for FQ, Fig. 2) did not significantly affected ACE in the lungs, indicating the absence of the captopril-specific side effect. It is expected that FBPs should have high safety and thus, they can be used as functional food or food supplements that may be effective in primary prevention of hypertension.

## Conclusion

We found that the main BPL mechanism of FBPs was ACE inhibition in living body tissue. Additionally, we suggest that our novel *ex vivo* assay was effective in evaluation of ACE-inhibitory mechanism with BPL action of peptidic ACE inhibitors in living body tissue.
